# Breast cancer risk by age at birth, time since birth and time intervals between births: exploring interaction effects

**DOI:** 10.1038/sj.bjc.6602302

**Published:** 2004-12-14

**Authors:** G Albrektsen, I Heuch, S Hansen, G Kvåle

**Affiliations:** 1 Department of Mathematics, University of Bergen, Johannes Brunsgt. 12, 5008 Bergen, Norway; 2 Cancer Registry of Norway, Montebello, 0310 Oslo, Norway; 3 Center for International Health, University of Bergen, Armauer Hansens hus, 5020 Bergen, Norway

**Keywords:** breast cancer, pregnancies, age at births, time since births, birth interval

## Abstract

In a Norwegian, prospective study we investigated breast cancer risk in relation to age at, and time since, childbirth, and whether the timing of births modified the risk pattern after delivery. A total of 23 890 women of parity 5 or less were diagnosed with breast cancer during follow-up of 1.7 million women at ages 20–74 years. Results, based on Poisson regression analyses of person-years at risk, showed long-term protective effects of the first, as well as subsequent, pregnancies and that these were preceded by a short-term increase in risk. The magnitude and timing of this adverse effect differed somewhat by birth order, maternal age at delivery and birth spacing. No transient increase in risk was seen shortly after a first birth below age 25 years, but an early first birth did not prevent a transient increase in risk after subsequent births. In general, the magnitude of the adverse effect was strongest after pregnancies at age 30 years or older. A wide birth interval was also related to a more pronounced adverse effect. Increasing maternal age at the first and second childbirth was associated with an increase in risk in the long run, whereas no such long-term effect was seen with age at higher order births.

From the numerous studies of reproductive history and breast cancer risk, there is a consensus that an early first birth and increasing number of full-term births are associated with a long-term reduction in risk (National Cancer Institute, 2003). However, a transient increase in risk after first birth has also been found, with a peak in risk within 5 years after delivery (Pathak, 2002; National Cancer Institute, 2003). This pattern may be due to a growth enhancing effect of oestrogens during pregnancy on premalignant breast cells (Henderson and Bernstein, 1991). It is likely that the susceptibility of the breast tissue cells increase with age. Thus, an adverse effect may be more pronounced after a pregnancy at an older age. However, few studies have investigated whether the risk pattern after pregnancy differs by maternal age at the childbirth, or whether subsequent pregnancies exert an independent adverse effect and whether birth spacing affects the risk pattern.

The present study aimed to obtain detailed information on associations between breast cancer risk and timing of births based on follow-up information for 1 691 555 Norwegian women of parity 0–5, including 22 890 breast cancer cases at ages 20–74 years.

## MATERIAL AND METHODS

The present study includes all Norwegian women born in the period 1925 to 1979, who had been residents of Norway for some period after 1960 and thus were included in the Norwegian Population Registry. The present data set is an update (until 1 January 2000) and extension of our previous data set (Albrektsen *et al*, 1994, 1995) with information on reproductive history (date of birth for each live born child) and breast cancer. Detailed information on the linking between data from national registers has been given previously (Albrektsen *et al*, 1994).

Each woman was considered at risk of breast cancer from age 20 years. However, since the Cancer Registry of Norway was established in 1953, no women entered the risk set before 1955. Thus, women born in 1925–1934 entered the risk set at age 21–30 years. At closing date of study, the oldest women were 74 years old. A total of 1 691 555 women of parity 0–5 were included in follow-up, contributing a total of 3788 × 10^5^ person-years in the age interval 20–74 years. The mean follow-up time was 24.9 years, range 1 month to 54 years.

Only cases classified as primary malignant neoplasms of the breast (ICD 7th Revision, 170) were considered. A total of 22 890 women were diagnosed with breast cancer during follow-up, supported by histological examination and/or by autopsy for 22 666 (99%). Of these, 22 510 (99.3%) were classified as carcinomas, 107 (0.5%) as sarcomas and 49 (0.2%) as other or unspecified tumours.

### Statistical analysis

Associations between breast cancer incidence and the reproductive factors were examined in a log-linear Poisson regression model of person-years at risk (Breslow and Day, 1987). To circumvent a collinearity problem in the analyses of joint effects of age at, and time since, the most recent birth in an age-adjusted model, the general age effect was estimated on the basis of nulliparous women (Albrektsen *et al*, 1995, 1999; Heuch *et al*, 1999, 2000). The association with attained age (1-year intervals, 20–74 years) was modelled as a cubic polynomial with terms age, (age)^2^ and (age)^3^, which gave a very good fit to the observed age-specific incidence rates among nulliparous women. Stratification was made on birth cohort (11 five-year categories).

Parity was treated as a time-dependent categorical variable, represented by indicator variables in the model. A woman entered a new exposure category (higher parity group) whenever she gave birth to a child. Only women of parity 5 or less were considered. Nulliparous women were assigned a constant value of age at births (corresponding to the chosen reference groups) and time since birth (corresponding to time zero, i.e. immediately after birth) to make it possible to estimate the age effect on the basis of this group (Heuch *et al*, 1999). Owing to the lack of variation, nulliparous women will not contribute to the estimation of risk estimates for the reproductive factors among parous women. The interpretation of the risk estimates for parity, however, will depend on which values are assigned to nulliparous women.

Time-dependent variables were defined also for age at each childbirth, with a constant reference value assigned until date of birth, and actual age at date of delivery otherwise. In this way, the terms for age at *N*th birth only entered the expressions for risk in women with at least *N* births. Age at each birth was treated either as a continuous variable in 1-year intervals (when testing for linear trend), or as a categorical variable (5-year groups). Among women with five full-term births, only 5-year categories were considered (midpoint assigned when treated as interval variable).

A restricted cubic spline curve (Durrleman and Simon, 1989) was used to allow for a nonlinear association with time since the most recent birth (time-dependent variable) in 1-year intervals (knots at 1, 5, 10, 15 and 25 years after birth). The curve is constrained to be linear at the extremes, that is, below the first and above the last knot. With five knots, only four parameters (one linear, three cubic terms) were necessary for modelling the association.

In joint analyses of age at, and time since, a particular childbirth, adjustment was made for age at all previous births. Owing to collinearity, the estimated associations with age at previous births will partly reflect relations with time since previous births. Interaction terms were examined whether the association with time since birth, as represented by the spline regression coefficients, differed by order of birth, age at delivery or birth interval. Based on the values of the estimated regression coefficients, combined with coefficients for parity and age at births, the difference in estimated log-rate between a particular subgroup of parous women and nulliparous women over time was calculated. An antilogarithmic transformation was made to determine incidence rate ratios. For particular purposes, the incidence rate according to attained age was calculated, with time-dependent contribution of the reproductive factors.

Tabulation of individual records into person-years tables and Poisson regression analyses of person-years at risk, with calculation of maximum likelihood estimates and likelihood ratio tests, were performed by means of the EPICURE program package (Preston *et al*, 1996).

## RESULTS

Table 1 shows the distribution of cancer cases and person-years according to reproductive factors. The mean ages (standard deviation) of mothers at the first to fifth childbirth were 24.2 (4.4), 27.0 (4.4), 29.5 (4.6), 31.2 (4.7) and 32.6 (4.6) years, respectively. The total number of children decreased over time (means of 2.3, 2.1, 1.9 and 1.6 for birth cohorts <1940, 1940–1949, 1950–1959 and 1960–1969, respectively).

### Mother's age at first and subsequent births

Since mutual adjustment can be made only for age at and time since the most recent birth, an association with age at *N*th birth can be assessed properly only among women of parity *N*. In particular, the association with age at first birth can be properly examined only among uniparous women. In fact, we found a considerably weaker estimated association with age at the first birth among uniparous (Table 2, 5% increase in risk per 5-year increase in age) than multiparous women (Table 2, 9–16% per 5-year). Thus, the apparently stronger association among multiparous women probably reflects confounding by time since the first birth.

The association with age at second birth among biparous women was rather similar to the relation with age at first birth (Table 2). No significant overall association was found with age at the third or fourth births (Table 2). A stronger association with age at the third birth among women with more than three births may reflect confounding by time since the third birth. Owing to the few cancers, no separate analysis was performed for women with five births.

### Time since birth by parity

**Figure 1:**
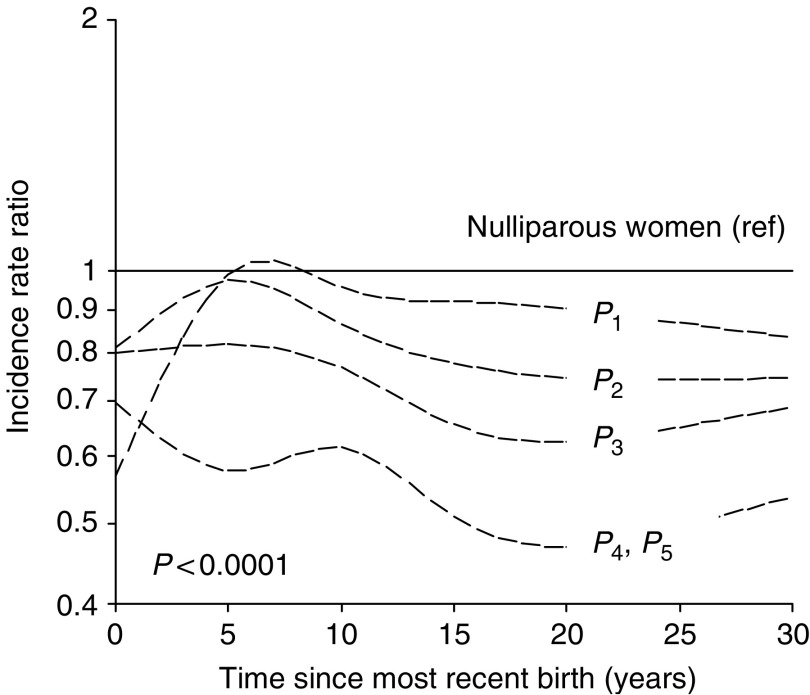
Predicted incidence rate ratio of breast cancer for women of parity *i* (*P*_*i*_, *i*=1–5, with a common effect of parity 4–5) according to time since *i*th birth, relative to nulliparous women. Results are adjusted for age, birth-cohort, age at *i*th, that is, most recent, and all previous births (assuming a common effect of age at *i*th birth among women of parity ⩾*i*), and with interaction between parity and time since birth. The predicted risk level corresponds to reference categories of ages at births, that is, the youngest categories of age at *N*th birth (*N*=1–5) shown in Table 1.

The estimated spline regression coefficients, with corresponding predicted risk curves for time since the most recent birth according to order of birth (relative to nulliparous women) are shown in Table 3 and Figure 1, respectively. The maximum follow-up ranged from 43 to 54 years after birth. The predicted risk is shown for the first 30 years, with a linear relation after 25 years.

Each additional child contributed to an overall reduction in risk. Without considering interaction effects, the incidence rate ratios (with 95% CI) for women with two to five full-term births *vs* uniparous women were 0.91 (0.85–0.96), 0.81 (0.73–0.89), 0.64 (0.57–0.72), and 0.50 (0.38–0.66), respectively. However, the association with time since birth differed by birth order (*P*<0.0001 in an overall test for difference in the spline regression coefficients between parity groups, Table 3), making it difficult to interpret overall effects of parity. The significant interaction appeared to be explained by differences in the risk pattern shortly after delivery since the risk curves were almost parallel after about 15 years (Figure 1). The adverse effect seemed to be strongest after the first birth, with a peak in risk about 6.5 years after birth. The very low estimated risk immediately after delivery may be related to the linear constraints in the first part of the smoothed cubic spline curve, combined with a subsequent rapid increase in risk. A transient increase in risk was seen also after the second birth, with a maximum after about 5 years. No increase in risk was observed after the third birth, but a delayed adverse effect appeared after the fourth and fifth births, with a maximum about 10 years after delivery. The association with time since the most recent birth, as represented by the linear and nonlinear cubic spline regression coefficients, was significant at the 5% level for all births, except the fifth (Table 3). Analyses within each parity group (first to third births only) were performed to explore interaction effects with age at birth and birth spacing.

### Time since birth by age at delivery and birth spacing

#### Uniparous women

**Figure 2:**
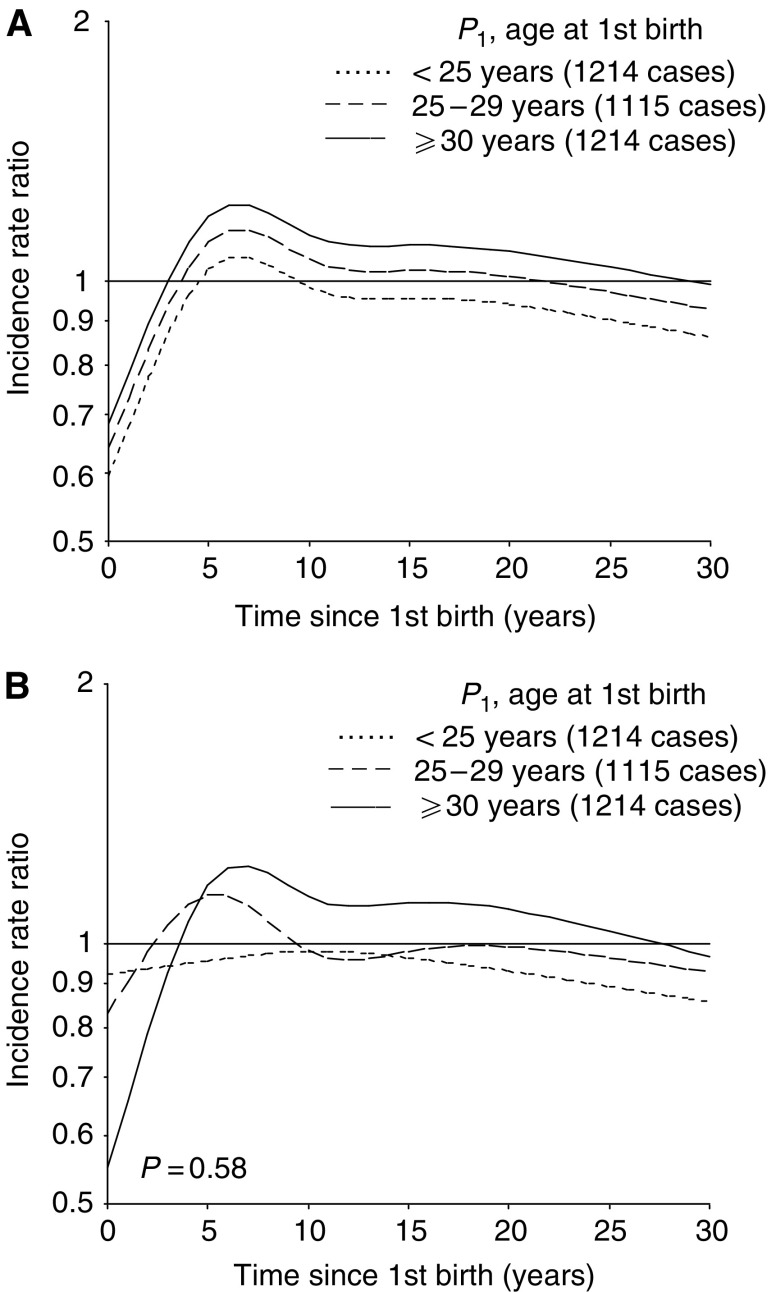
Predicted incidence rate ratio of breast cancer for uniparous women (*P*_1_) according to time since first birth in subgroups of age at first birth, relative to nulliparous women, calculated on the basis of a model (**A**) without and (**B**) with, interaction between age at and time since the first birth. Results are adjusted for age and birth-cohort.

Figure 2 shows the predicted risk of breast cancer according to time since first birth by age at the birth, calculated on basis of a model without and with an interaction effect (Figure 2A and B, respectively). The risk pattern after the first birth seemed to depend on the mother's age at delivery, although the interaction test did not reach statistical significance (*P*=0.58). Based on the interaction model (Figure 2B), there was no increase in risk after an early first birth (<25 years). The adverse effect was strongest after a late first birth (⩾30 years). About 15 years after delivery, the risk curves were rather parallel, but with a consistent difference in risk according to age at first birth. In comparison to nulliparous women, uniparous women with an early first birth (<25 years) always had a lower risk, whereas a higher risk was seen until 10–30 years after delivery among women with a first birth at an older age.

#### Biparous women

**Figure 3:**
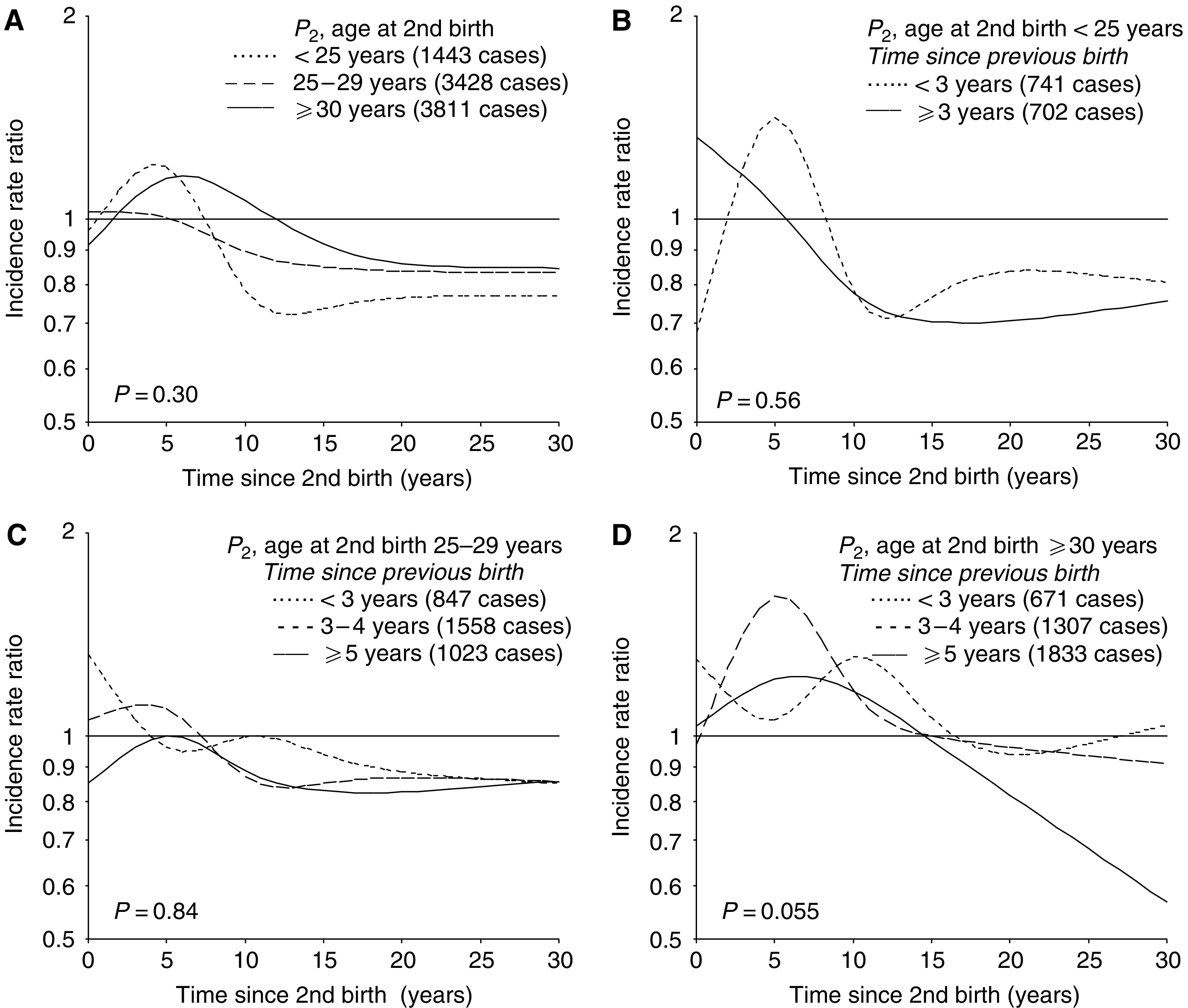
Predicted incidence rate ratio of breast cancer for biparous women (*P*_2_) according to time since second birth in (**A**) subgroups of age at the second birth (interaction model), and for each category of age at second birth, that is, (**B**) <25, (**C**) 25–29, and (**D**) ⩾30 years, within subgroups defined by time interval between first and second birth, relative to nulliparous women. Results are adjusted for age and birth-cohort, and in (**A**) also for age at the first birth.

The youngest biparous mothers (second birth <25 years) experienced a transient increase in risk shortly after birth, whereas no adverse effect appeared among women with a second birth at age 25–29 years (Figure 3A). Among the oldest mothers (second birth ⩾30 years), the adverse effect was more long-lasting than in the youngest group. The risk curves were rather parallel after about 15 years, but with an overall difference in risk between those with a second birth below and above the age of 25 years. The shape of the risk curves did not differ significantly by age at the birth (*P*=0.30).

Additional subgroup analyses showed that the sharp transient increase in risk among the youngest mothers was restricted to those with first and second birth close in time (<3 years, Figure 3B). This pattern may reflect a continuation of the risk pattern after the first birth. A moderate transient increase in risk was now found also after a second birth at age 25–29 years, but timing of the adverse effect varied according to birth spacing (Figure 3C). Birth spacing had a similar effect among the oldest biparous women (Figure 3D, *P*=0.055). In general, a narrow birth interval (<3 years) seemed to result in a high initial risk followed by a delayed adverse effect, whereas a wide birth interval was associated with a faster and more long-lasting elevation in risk.

#### Triparous women

**Figure 4:**
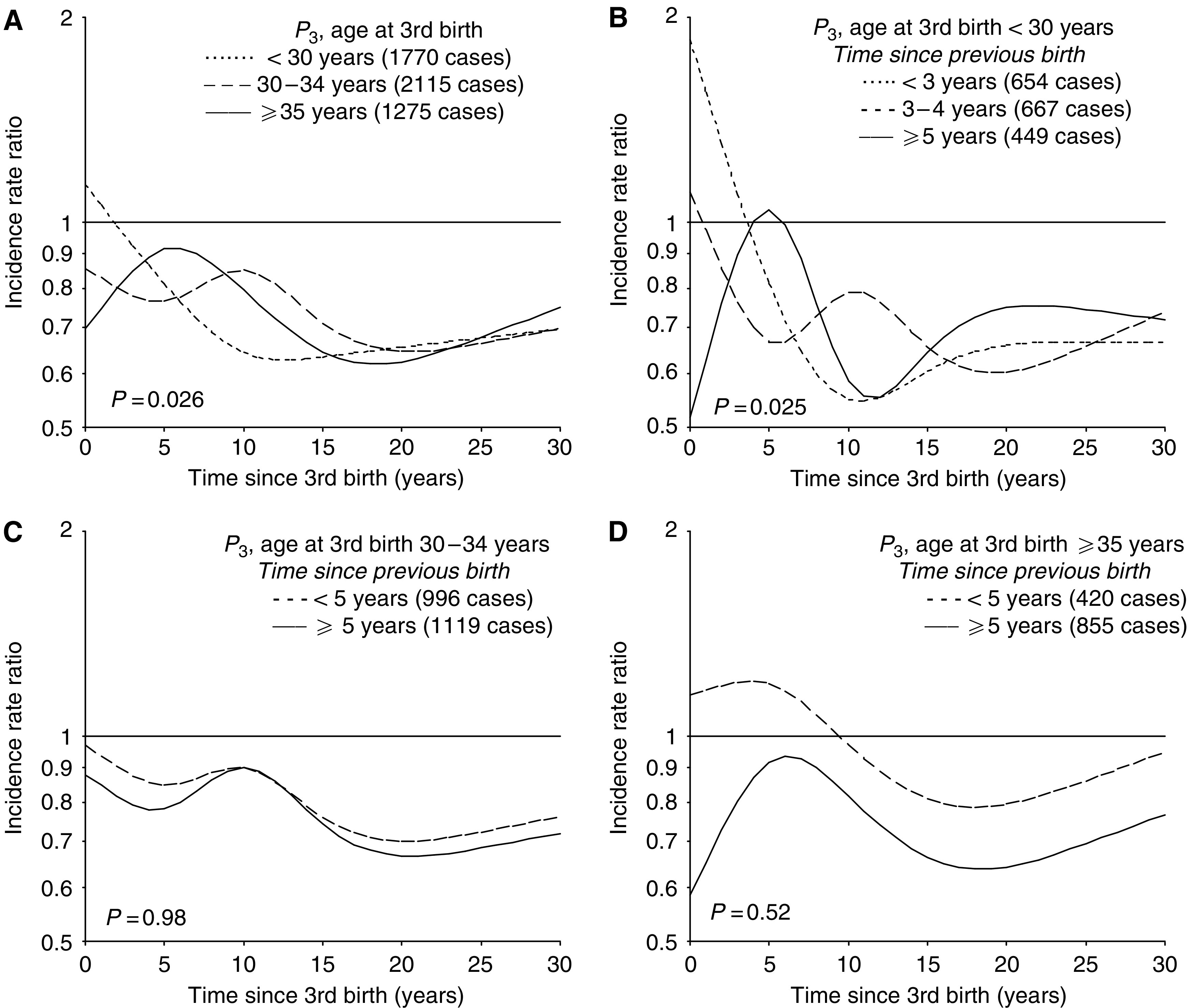
Predicted incidence rate ratio of breast cancer for triparous women (*P*_3_) according to time since third birth in (**A**) subgroups of age at the third birth (interaction model), and for each category of age at third birth, that is, (**B**) <30, (**C**) 30–34, and (**D**) ⩾35 years, within subgroups defined by time interval between second and third birth, relative to nulliparous women. Results are adjusted for age, birth-cohort, age at first birth and in (**A**) also for age at the second birth.

The risk pattern after third birth differed significantly by the mother's age at delivery (Figure 4A, *P*=0.026). A transient increase in risk was seen among women with a third birth at age ⩾30 years only, but with an earlier peak in risk among the oldest mothers (⩾35 years). Age at the third birth seemed to be of less importance for the long-term risk, as the risk curves in the different subgroups appeared to overlap after some time.

Further analyses according to birth spacing showed a transient increase in risk also after a third birth at a younger age (<30 years), unless the second birth had occurred within the last 3 years (Figure 4B). Among the oldest mothers, the risk pattern after the third birth did not differ according to birth spacing (Figure 4C, D).

### Absolute risk by attained age, parity, age at births and time since births

**Figure 5:**
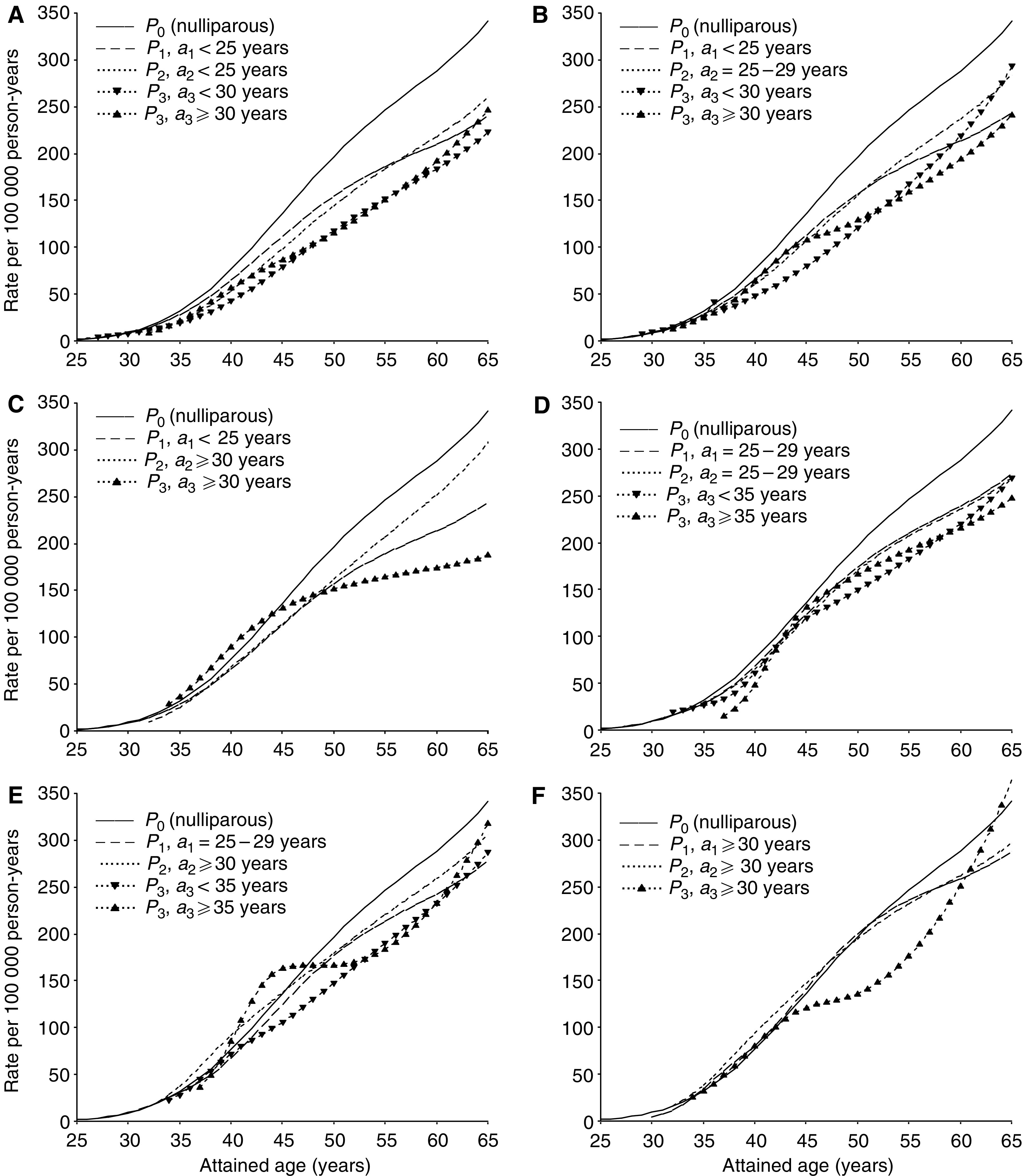
Predicted incidence rate of breast cancer for women of parity 0–3 (*P*_0_, *P*_1_, *P*_2_ and *P*_3_, respectively) by attained age, with additional contribution from each subsequent child according to time since the most recent birth, in specific combinations of ages at first, second and third birth (*a*_1_, *a*_2_, and *a*_3_), respectively. (**A**)–(**C**) represent combinations with an early first birth, (**D**), (**E**) a first birth at an intermediate age, and (**F**) a late first birth. Results are adjusted for age and birth-cohort.

Figure 5A–F shows the predicted incidence of breast cancer according to attained age in subgroups defined by parity, age at birth and time since birth. Each figure shows the risk pattern for women of parity 0–3, with the same ages at births. Thus, these curves illustrate the effect of having an additional child at different ages. The prediction is based on a model with age at births in 5-year categories. When a single birth occurs within a specific age-category, the contribution to risk starts at the midpoint in the age interval. Otherwise, the first birth starts contributing at the lowest age and the subsequent births at 2-year intervals.

#### Short-term effects in terms of absolute risk

In view of the low incidence rate at young ages, the transient increase in relative risk after a childbirth before age 30 years almost disappeared in terms of changes in absolute rates (Figure 5A–E). However, the adverse effect was quite evident after a pregnancy at age 30 years or older, regardless of order of birth. In certain combinations of age at births, the transient increase in risk seemed to be strengthened for each additional child (Figure 5C–E). Of particular interest was the observation that an early first birth did not prevent an adverse effect of subsequent births at a high age (Figure 5A-C). However, both age at the birth as well as birth spacing, were important for the magnitude of the adverse effect (Figure 5A *vs* B, 5D *vs* E and 5C *vs* F). The transient increase in risk after a third birth was particularly strong among triparous women who had their first birth at age 25–29 years, with a wide birth spacing between the first and the two subsequent births (Figure 5E).

#### Long-term effects in terms of absolute risk

Consistent with the relative risk estimates, uniparous women always reached a lower risk level than nulliparous women in the long run (Figure 5A–F). Among women with an early first birth (Figure 5A–C), the protective effect of the second pregnancy became weaker with increasing age of the mother at time of the delivery. Among women with a first birth at an older age, there was no large difference in long-term risk between uni- and biparous women (Figure 5D–F). A third birth was associated with an additional long-term reduction in risk, regardless of age at the birth (Figure 5A–F). The apparent increase in risk in the last part of follow-up in some subgroups is probably related to more sparse data together with the linear constraint imposed after the last knots in the spline regression curve.

## DISCUSSION

In this large population-based study, we have explored time-related effects of pregnancies on breast cancer risk. The data were based on compulsory registrations in nationwide registers, and are thus not influenced by selection or recall bias. Some misclassification of parity might have occurred, in particular for women born before 1935 (Brunborg and Kravdal, 1986; Albrektsen *et al*, 1995). However, the number of births was rather similar for cohorts 1925–1934 and 1935–1939. The size of the data set made it possible to explore interaction effects between correlated reproductive factors.

In general, it is difficult to distinguish between the effect of age at and time since a particular childbirth as all women at the same age with a specific age at birth will have exactly the same value of time since the birth. Consequently, it is impossible to perform an age-adjusted analysis with mutual adjustment for these two reproductive factors among parous women (Albrektsen *et al*, 1995; Leon *et al*, 1995; Hsieh and Lan, 1996; Cummings *et al*, 1997; Heuch *et al*, 1999, 2000). However, it is possible to circumvent the collinearity problem by estimating the general age effect on the basis of nulliparous women, assuming that the variation in breast cancer risk due to ageing is the same for nulliparous and parous women (Albrektsen *et al* 1995, 1999; Heuch *et al*, 1999, 2000). With sufficiently large data sets, this method provides reliable and unbiased risk estimates (Albrektsen *et al*, 1999). It is not possible, however, to investigate interaction effects with age in this model. Some previous studies have focused on a potential interaction between parity and age. The apparent unfavourable effect of high parity among women below the age of 45–50 years, in contrast to the protective effect among older women, may be explained by a transient increase in risk shortly after birth. Such an effect can be expressed more directly through an association with time since birth. Focusing on the joint effect of all births introduces additional collinearity problems that also involve birth spacing. Expressing the model in terms of particular variables makes it possible to explore different questions of interest, although it is difficult to sort out which variables are most important.

We applied a restricted cubic spline function for representing the nonlinear relationship with time since a childbirth. A cubic spline is a piecewise polynomial that is visually smooth owing to the constraints at the join points, or knots (Durrleman and Simon, 1989). A main advantage of a restricted cubic spline function is that quite an arbitrary nonlinear risk pattern can be fitted with a limited number of parameters, even with time since birth in 1-year intervals. This also makes it easier to test for interaction effects. In our study, the selection of knots (number and positions) was adapted to the range in follow-up after birth in our data, and also to prior knowledge about the risk pattern. Alternative knots were applied (i.e. 4 or 5 knots, different positions), but quite similar results were achieved. In certain subgroups, however, a model with four knots gave results that were very sensitive to the location of knots, in contrast to those obtained with five knots. The final results were based on spline curves with five knots.

Consistent with results from a recent large study that applied a similar analytical approach (Liu *et al*, 2002), we observed an increase in risk of breast cancer with increasing age at first birth above what could be explained by a time delay in the protective effect of the pregnancy, that is, by a decrease in risk with increasing time since the birth. We also found that the transient increase in risk shortly after the birth was strongest after a late first birth. Liu *et al* (2002) did not find any significant interaction and did not calculate separate risk estimates. However, it is difficult to demonstrate significant differences when the risk curves are similar in shape, and only a restricted part of the total curve differs. The power of the test may also be low in the analyses for uniparous women (Albrektsen *et al*, 1999).

We found that uniparous women with a late first birth (⩾30 years) had a higher risk than nulliparous women until about 27 years after delivery, that is, at an age of at least 57 years. With a first birth at a lower age, the risk curves crossed earlier. In terms of absolute rate, the predicted crossover point was somewhat earlier due to some variation in the age estimates between the different analyses. Based on the ‘breast-tissue age’ model (Pike *et al*, 1983), Rosner and Colditz (1996) found that uniparous women with a first birth at age ⩾30 years had higher risk at ages 55–64 years, whereas women with an earlier first birth had a lower risk. Women with a first birth at age ⩾35 years always had a higher risk (Rosner and Colditz, 1996; Colditz and Rosner, 2000), but the predictions for this subgroup were based on sparse data. Other studies have reported a different crossover point according to age at the first birth, either in terms of attained age or time since the birth (Hsieh *et al*, 1994; Lambe *et al*, 1994; Robertson *et al*, 1997; Chie *et al*, 2000; Wohlfahrt and Melbye, 2001; Liu *et al*, 2002). In most of these studies, however, all uniparous women eventually had a lower risk. In two studies (Lambe *et al*, 1994; Robertson *et al*, 1997) women with a late first birth had the lowest long-term risk (but highest risk shortly after birth), but this pattern appeared to be a consequence of linear constraints in the model. Our results, based on quite detailed modelling, indicate that a late first birth is also associated with a long-term protective effect, despite a more pronounced adverse effect in the first years after the delivery.

We found an association with age at the second birth among biparous women with long-term risk differing by second birth below and above age 25 years. The few studies that found an association with age at second birth (Negri *et al*, 1990; Lambe *et al*, 1994; Wohlfart and Melbye, 2001) did not adjust for time since the birth. In one study that did so (Liu *et al*, 2002), only a weak association was found. We found a somewhat inconsistent pattern between uni- and biparous women, possibly due to an independent adverse effect of the second pregnancy, combined with an effect of age at the births. Nevertheless, the long-term protective effect appeared to be more pronounced when both births were at an early age. A less pronounced protective effect of a second child subsequent to a late first birth has been reported by others (MacMahon *et al*, 1982; Trichopoulos *et al*, 1983, Decarli *et al*, 1996). Compared to all uniparous women, some studies (Lambe *et al*, 1994; Hsieh and Lan 1996; Liu *et al*, 2002) have found a higher risk shortly after birth among women with a second birth at age at 35 years or older, but a lower or rather similar risk in the long run. One study (Chie *et al*, 2000) reported a protective effect of the second child regardless of age at the birth. Overall, the mother's age at the second birth certainly appears to influence the subsequent risk of breast cancer. In view of time-related effects, as well as dependency on the timing of the first birth, however, it is difficult to interpret risk estimates for an overall association with age at the second birth.

Consistent with results of previous studies (Trichopoulos *et al*, 1983; Decarli *et al*, 1996; Robertson *et al*, 1997; Chie *et al*, 2000, Wohlfahrt and Melbye, 2001), we observed only a weak overall association with the mother's age at third or higher order births. Wohlfart and Melbye (2001) found a more pronounced association with age at the third birth among women with 10 or more years since the birth. Taking into account effect modification by age at the birth, our study revealed a short-term adverse effect also of the third pregnancy. Differentiation of breast cells after first full-term birth is assumed to make breast tissue less vulnerable to cancer and thus result in a less pronounced or even no adverse effect of subsequent births (Pathak, 2002). In our study, an early first birth did not prevent an adverse effect of the third pregnancy. We are not aware of other studies that have explored time-related effects of third or higher order births.

An association between mitotic activity of the breast tissue and hormonal factors, in particular those related to oestrogens and prolactin levels, but possibly also progesterone, was a main concept underlying the breast-tissue age model of Pike *et al* (1983). In accordance with such a mechanism, elevated levels of oestrogens during pregnancy have been suggested to act as a promotor on premalignant breast cells and thus explain the transient increase in risk after delivery (Henderson and Bernstein, 1991; Pathak, 2002). The oestrogen level is higher in the first than subsequent pregnancies (Bernstein *et al*, 1986), consistent with previous observations of a more pronounced adverse effect of the first birth (Pathak, 2002). In the present study, however, a similar adverse effect was found also after subsequent pregnancies after age 30 years. The proportion of oestrogen and progesterone receptor positive breast tumours has been found to increase with age (Habel and Stanford, 1993; Colditz *et al*, 2004), possibly making a potential promoting effect of pregnancy oestrogens more pronounced, regardless of birth order. One study (Walker *et al*, 1996) found the highest proportion of these receptors at age 35–39 years. In our study, the increase in absolute risk was particularly pronounced after a third birth at this age, supporting the hypothesis of a promoting effect of pregnancy hormones, although not necessarily of oestrogens. A higher proportion of oestrogen-negative tumours has been reported among women with a recent childbirth, who were mainly in the age group 30–39 years (Phillips *et al*, 2004). Another study found a transient increase in risk after first birth mainly in progesterone receptor negative tumours, regardless of oestrogen receptor status (Colditz *et al*, 2004). Thus, other mechanisms for explaining the transient increase in risk shortly after birth are possible.

In summary, the present study showed a rather complex association between reproductive history and breast cancer risk. The mother's age at births, as well as birth spacing, influenced the magnitude and timing of the transient increase in risk shortly after a delivery. In the long-term, however, only the number of births and the mother's age at the first and second birth seemed to be important for the risk level. The changes in childbearing pattern during recent decades, with fewer children and higher age at births (Lee *et al*, 2003), will probably affect cancer incidence in the future. Long-term follow-up data of the youngest birth-cohorts are needed to fully explore the effect of childbirth late in reproductive life.

## Figures and Tables

**Table 1:** Number of breast cancer diagnoses (no. of cases) and person-years (P-yr) by reproductive factors during follow-up of 1.7 million Norwegian women aged 20–74 years

	**Total**		**1st birth**		**2nd birth**		**3rd birth**		**4th birth**		**5th birth**	
	**No. of cases**	**P-yr (× 10^4^)**	**No. of cases**	**P-yr (× 10^4^)**	**No. of cases**	**P-yr (× 10^4^)**	**No. of cases**	**P-yr (× 10^4^)**	**No. of cases**	**P-yr (× 10^4^)**	**No. of cases**	**P-yr (× 10^4^)**
*Parity* a												
0	3306	1066.1										
⩾1	19 584	2722.3	3543	698.0	8682	1125.6	5160	619.4	1757	213.6	442	65.7
												
*Time since most recent birth (years)* b												
<5	1403	981.9	232	357.0	582	373.3	402	178.9	152	56.1	35	16.5
5–9	2144	503.0	307	110.4	936	219.3	626	121.6	217	39.9	58	11.8
10–14	2749	365.5	415	65.9	1183	161.4	794	94.8	280	33.1	77	10.3
15–19	3297	295.7	540	50.5	1536	129.8	865	77.6	282	28.5	74	9.1
20–24	3318	233.7	591	39.9	1534	99.7	835	62.1	296	24.2	62	7.8
25–29	2978	166.6	543	29.6	1273	68.4	797	45.2	299	18.3	66	5.1
30–34	2010	99.6	374	20.4	885	41.8	560	25.9	169	9.8	22	1.7
35–39	1053	49.5	282	13.4	506	22.3	215	10.4	48	3.0	2	0.2
⩾40	586	23.6	259	10.8	247	9.6	66	2.7	14	0.5	0	0.005
												
*Mother's age at delivery (years)*^c^,^d^												
<20			2164	468.6	—	—	—	—	—	—	—	—
20–24			8574	1358.6	4229	772.7	713	138.3	—	—	—	—
25–29			6034	681.3	6648	836.8	2423	362.6	524	98.5	56	12.8
30–34			2065	170.5	3805	334.8	2786	292.2	838	110.0	149	24.6
35–39			747	43.3	1177	71.5	1248	93.9	689	60.2	179	22.0
⩾40			—	—	182	8.7	189	11.7	148	10.5	58	6.3

a Number of full-term births.

b The total number in first column is based on women with at least one birth.

c The numbers for age at *N*th birth (*N*=1–5) are based on women with at least *N* births.

d Categories marked with ‘—’ are combined with category below (older mothers) or above (younger mothers), respectively.

**Table 2:** Incidence rate ratios (IRR with 95% confidence intervals) for age at *N*th birth (*N*=1–4) within each parity group^a^

	**Unparous women**		**Biparous women**		**Triparous women**		**Quadriparous women**	
	**No. of cases**	**IRR (95% CI)**	**No. of cases**	**IRR (95% CI)**	**No. of cases**	**IRR (95% CI)**	**No. of cases**	**IRR (95% CI)**
*Age at 1st birth (years)*								
<25 years	1214	1.00 (ref)	4478	1.00 (ref)	3392	1.00 (ref)	1302	1.00 (ref)
25–29 years	1115	1.08 (0.99–1.17)	2982	1.08 (1.02–1.14)	1468	1.09 (1.01–1.18)	387	1.05 (0.91–1.22)
30–34 years	749	1.14 (1.03–1.26)	976	1.12 (1.02–1.23)	268	1.11 (0.95–1.28)	68	1.22 (0.88–1.67)
⩾35 years	465	1.18 (1.04–1.34)	246	1.29 (1.11–1.51)	32	—b		—b
IRR for trend (per 5 year)		1.05 (1.02–1.09)		1.09 (1.05–1.14)		1.09 (1.02–1.18)		1.16 (1.00–1.35)
*Age at 2st birth (years)*								
<25 years			1443	1.00 (ref)	1720	1.00 (ref)	811	1.00 (ref)
25–29 years			3428	1.08 (1.01–1.16)	2320	1.05 (0.98–1.13)	739	1.04 (0.92–1.18)
30–34 years			2649	1.15 (1.06–1.25)	946	1.20 (1.07–1.35)	207	0.97 (0.76–1.23)
⩾35 years			1162	1.22 (1.09–1.36)	174	—b		—b
IRR for trend (per 5 year)				1.04 (1.00–1.09)		1.05 (0.97–1.13)		0.94 (0.78–1.12)
*Age at 3st birth (years)*								
<30 years					1770	1.00 (ref)	1027	1.00 (ref)
30–34 years					2115	1.04 (0.96–1.12)	580	1.04 (0.91–1.20)
⩾35 years					1275	1.05 (0.95–1.16)	150	1.20 (0.94–1.52)
IRR for trend (per 5 year)						1.02 (0.97–1.07)		1.06 (0.94–1.20)
								
*Age at 4st birth (years)*								
<30 years							336	1.00 (ref)
30–34 years							649	1.08 (0.93–1.24)
⩾35 years							772	1.27 (1.07–1.50)
IRR for trend (per 5 year)								1.05 (0.97–1.15)

a Results based on Poisson regression analyses, with adjustment for age, birth-cohort, time since the most recent birth and age at all previous births.

b Combined with category above.

**Table 3:** Cubic spline regression coefficients (with *P*-values) for association with time since the most recent birth among women with 1–5 full-term births^a^

	**1st birth**	**2nd birth**	**3rd birth**	**4th birth**	**5th birth**
*Time since most recent birth (tsb)*					
Linear term (tsb⩾0)	0.1329 (<0.001)	0.04844 (0.03)	0.006922 (>0.5)	−0.07091 (0.09)	0.04596 (>0.5)
1st cubic term (tsb>1, 15, 25)^b^	−0.001693 (0.005)	−0.000873 (0.018)	−0.0001609 (>0.5)	0.001121 (0.13)	0.0000395 (>0.5)
2nd cubic term (tsb>5, 15, 25)^b^	0.003394 (0.014)	0.001748 (0.036)	0.0000742 (>0.5)	−0.002754 (0.10)	−0.001011 (>0.5)
3rd cubic term (tsb >10, 15, 25)^b^	−0.002093 (0.066)	−0.0009613 (0.16)	0.0005423 (>0.5)	0.002705 (0.059)	0.002402 (0.4)
					
IRR for trend (per year)	−0.0039	−0.00633	−0.00449	−0.00514	−0.00444
*P, test for trend* c	0.05	0.0004	0.029	0.089	0.43
*P, test for deviation from trend* d	<0.0001	0.0001	<0.0001	0.0022	0.085

a Results based on Poisson regression analyses, with adjustment for age, birth-cohort, parity, age at most recent and all previous births (categorical, 5-year intervals) in a model with interaction between parity and time since the most recent birth.

b Nonlinear terms in the cubic spline regression equation are added as time, in terms of time since birth (tsb), increases, depending on the values of knots (1, 5, 10, 15, 25).

c Test for linear term in the spline regression equation before cubic terms are added, corresponding to ordinary test for trend (and IRR for trend).

d Test of significance of contribution of all three cubic terms, that is, the nonlinear part, in the spline regression equation.
